# 68127 High Sensitivity Troponins Predicts Mortality in Patients Who Present to the ED with Severe Sepsis or Septic Shock

**DOI:** 10.1017/cts.2021.485

**Published:** 2021-03-30

**Authors:** Kendrick Williams, Ryan Tucker, James Cranford, Christopher Fung

**Affiliations:** 1 University of Michigan Medical School; 2 Michigan Medicine Department of Emergency Medicine

## Abstract

ABSTRACT IMPACT: Our may suggest that delta hsTrop could be of prognostic value in patients with sepsis. OBJECTIVES/GOALS: - METHODS/STUDY POPULATION: We analyzed data of those presenting to the ED over an 18-month period with sepsis and at least one episode of hypotension after 1 liter of IV fluids. We performed a retrospective analysis using a cohort derived from modified inclusion and exclusion criteria from the CLOVERS study. The outcomes of patients found to have a delta (at least 6 pg/dL) in high sensitivity troponin T were compared to patients who did not have a delta or have a troponin level measured. We examined demographic and treatment characteristics of this cohort and the incidence of adverse outcomes were determined. We used multivariable logistic regression analysis to test the association of hsTrop and mortality. RESULTS/ANTICIPATED RESULTS: 778 patients met criteria to be included in the cohort. 279 patients had a change in high sensitivity troponins, an incidence of 35.9%. Patients with a delta were more likely to be older, male, and have a higher Charlson index than patients without a delta or those that had no troponin measured. They were also more likely to have a history of chronic lung disease, heart failure and hypertension. Change in high sensitivity troponins were associated with higher in-hospital mortality. When adjusted for age, gender, and Charlson Index, the association between a positive delta troponin and mortality remained statistically significant. DISCUSSION/SIGNIFICANCE OF FINDINGS: In patients with severe sepsis and septic shock, the presence of a positive or negative delta hsTrop at 2 hours is associated with increased mortality. Measurement of high sensitivity troponin early in the patient’s hospital course may have prognostic utility.

